# Summer Knitting

**Published:** 1917-04-14

**Authors:** John Penoyre

**Affiliations:** 8 King's Bench Walk, Inner Temple, E.C. 4.


### The Editor's Letter-Box.

### Summer Knitting.

*To the Editor of* The Hospital.

Sir,—In answer to countless letters on the question of what comforts to make for the men during the summer, can you find me room to state, on behalf of Sir Edward Ward, Director-General of Voluntary Organisations, that last year we over-estimated the interval between the last cold night of spring and the first chills of autumn, and that a vast store of sweaters, mufflers, helmets, mittens, and socks will be wanted by August 1? These should be sent, when the time draws near, either to the D.G.V.O.'s Depôt, 45 Horseferry Road, S.W. 1, or to me as below. Easily knitted patterns of any of the above can be had free on application, but, as the correspondence is necessarily heavy, a few stamps occasionally will be acceptable.

On behalf of the men, I have to thank your readers very gratefully for over 38,000 sweaters and 34,000 supplementary comforts already sent me.—Yours faithfully,

John Penoyre.

8 King's Bench Walk, Inner Temple, E.C. 4.
April 5, 1917.

